# Exploring patient and pharmacist perspectives on complex interventions for cardiovascular prevention: A qualitative descriptive process evaluation

**DOI:** 10.1111/hex.13133

**Published:** 2020-10-13

**Authors:** David J. T. Campbell, Terry Saunders‐Smith, Braden J. Manns, Marcello Tonelli, Noah Ivers, Brenda R. Hemmelgarn, Ross T. Tsuyuki, Raj Pannu, Kathryn King‐Shier

**Affiliations:** ^1^ Department of Community Health Sciences Cumming School of Medicine University of Calgary Calgary AB Canada; ^2^ Department of Medicine Cumming School of Medicine University of Calgary Calgary AB Canada; ^3^ Department of Cardiac Sciences Cumming School of Medicine University of Calgary Calgary AB Canada; ^4^ Department of Family & Community Medicine Faculty of Medicine University of Toronto Toronto ON Canada; ^5^ Department of Family & Community Medicine Women's College Hospital Toronto ON Canada; ^6^ Department of Medicine Faculty of Medicine & Dentistry University of Alberta Edmonton AB Canada; ^7^ Department of Pharmacology Faculty of Medicine & Dentistry University of Alberta Edmonton AB Canada; ^8^ Emergence Creative New York NY USA; ^9^ Faculty of Nursing University of Calgary Calgary AB Canada

**Keywords:** participant focus group, pharmacist, qualitative, randomized controlled trials, vulnerable population

## Abstract

**Background:**

The Assessing outcomes of enhanced Chronic disease Care through patient Education and a value‐baSed formulary Study (ACCESS) is a randomized controlled trial evaluating two interventions targeting barriers to care among those at high risk of cardiovascular disease: copayment elimination for cardioprotective medications, and a tailored self‐management support programme. We designed a process evaluation to better understand participant perspectives on the interventions.

**Design:**

We used a qualitative descriptive study design, collecting patient and pharmacist feedback via individual semi‐structured telephone interviews and in‐person focus groups. Data were analysed inductively using thematic analysis.

**Results:**

Fifty‐three patients (39 interviews and 14 in two focus groups) and 20 pharmacists participated. Copayment elimination provided quality of life benefits: minimizing the need to 'cut‐back', allowing 'peace of mind' and providing emotional support. Health‐related benefits included: improving adherence to covered medications, and helping to afford non‐covered goods. The only criticism was that not all medications and testing supplies were covered. Patients reported that the educational materials provided helpful information, acted as a reminder, improved confidence, improved adherence to medication, and helped initiate conversations with providers about indicated medication. Some participants felt that the educational materials were repetitive, overly medication‐focused and not tailored enough. Pharmacists felt that their patients benefitted from both interventions, which improved patient adherence and communication with their patients.

**Conclusion:**

The success of interventions intended to change behaviour is largely dependent upon participant's feelings that the intervention is helpful. This process evaluation provided insights into participants' perceptions on these interventions. Reception of both was largely positive with a few criticisms noted.

## 1 INTRODUCTION

Many patients at high cardiovascular (CV) risk have chronic conditions requiring them to engage in self‐management to achieve optimal outcomes.^1^ Self‐management often includes taking medications regularly, following specified diets, self‐monitoring and being physically active.^2^ Unfortunately, patients often face substantial barriers that impede their ability to self‐manage, pre‐disposing them to development or progression of cardiovascular disease (CVD).^3^ Two particularly common barriers include lack of knowledge regarding chronic conditions/CV risk, and how to manage those appropriately^4^; and financial barriers, which impede access to the prerequisites needed for optimal self‐management.^5^, ^6^, ^7^ Recent evidence suggests that addressing these barriers has the potential to improve patient outcomes. Specifically, in health‐care systems that require patients to pay for medications, reducing patients' financial barriers through policies such as copayment elimination has been demonstrated to improve adherence and reduce CV risk.^8^, ^9^, ^10^ In diverse settings, improving chronic condition knowledge through self‐management education has demonstrated promise in helping patients reduce CV risk,^11^, ^12^, ^13^ and is recommended by major international guidelines.^14^, ^15^, ^16^, ^17^ Conducting qualitative research along side interventional studies is recognized as being important to elucidate feedback on trial interventions and implementation processes.^18^, ^19^, ^20^ While there are high‐quality trials supporting the role of interventions to target financial and knowledge‐related barriers, qualitative studies about the implementation and acceptability of the interventions are not frequently reported in the published literature.

The **A**ssessing outcomes of enhanced **C**hronic disease **C**are through patient **E**ducation and a value‐ba**S**ed formulary **S**tudy, or ACCESS trial, is an on‐going factorial 2 × 2 pragmatic randomized controlled trial, which started in November 2015. The trial aims to evaluate the impact of two interventions targeting financial barriers and lack of knowledge, among low‐income seniors with high CV risk in Alberta, Canada.^21^, ^22^ The interventions are as follows: (1) a comprehensive tailored self‐management education and support (SMES) programme including facilitated relay of clinical information to participants' health‐care providers; and (2) elimination of copayments for select high‐value cardioprotective medications (comparing with standard care: copayments of 30% of drug costs, to a maximum of $25/medication/dispensation). We hypothesized that these interventions would result in improved medication adherence and health behaviour changes, ultimately resulting in fewer hospitalizations, CV events and deaths.

While our interventions were grounded in theory,^21^ their successful implementation was essential for changing participant behaviour. Therefore, we designed ACCESS as a hybrid effectiveness‐implementation trial (type 1)^23^ featuring a qualitative descriptive implementation study to help us assess participants' perspectives on the interventions. The objective was to assess the implementation of our interventions and learn how to better support behaviour change for CV risk reduction in order to make further modifications to the interventions for the rest of the trial. We explored participants' and recruiting pharmacists' perspectives on the strengths and limitations of the trial interventions.

## 2 METHODS

### 2.1 ACCESS trial

This process evaluation was a substudy nested within the ACCESS trial. Eligible participants were adults over 65 years with an annual household income <$50 000 who were at high risk of CV events (diagnosis of any one of: chronic kidney disease, coronary artery disease, heart failure or stroke; or at least two of: diabetes, high cholesterol, high blood pressure or smoking). Enrolment was conducted through a variety of sources, the largest of which was community pharmacists, starting in November 2015 and ending in September 2018.^22^ 4674 patient participants were randomized into one of the four groups (self‐management education, copayment elimination, both interventions or control). Follow‐up surveys and administrative health data are being used to collect data over a 3‐year period. The primary outcome of the ACCESS trial is to determine the effect of these novel interventions on relevant clinical endpoints (mortality, myocardial infarction, cerebrovascular events, need for revascularization and chronic disease‐related hospitalizations). The trial is on‐going, with continued follow‐up of patients happening through 2021. The qualitative work presented here was designed in the protocol to provide feedback on the study interventions and to allow for changes to the interventions to improve their acceptability to study participants.^21^ There were approximately 500 participants enrolled and randomized in the ACCESS trial at the time this qualitative work was launched, with roughly equal numbers between the four arms of the trial.

The SMES intervention, branded as MOXIE, was codeveloped by the ACCESS team and a marketing firm (EMERGENCE Creative). Both the branding and the fact that the messaging was provided by a fictional 'peer' (named Moxie) were designed to engender personalized patient engagement. Those enrolled in this arm of the trial received weekly 'postcards from a friend', colourful trifold mailers with graphics focused on various aspects of CV risk reduction. This strategy was used as it was hoped that by giving people physical objects to place around their homes, they may serve as subtle reminders for behaviour change. Participants who stated a preference for electronic communication during eligibility screening received regular emails and access to a personalized electronic platform in addition to the mailers. The details of the programme are reported elsewhere,^21^ but tailoring of health information was on the basis of a few specific variables collected at the baseline assessment: patient's individual CV risk factors, smoking status, current medication use, and patient's self‐reported barriers to medication adherence. MOXIE provided additional 'health tools' for patients including pedometers and health tracking books. Finally, MOXIE included a facilitated relay intervention whereby patients were sent letters regarding indicated medications, which were tailored based on the information provided at baseline. Patients were instructed to take the letters to their prescribing physicians and pharmacists to start a discussion about cardioprotective medications the patient should be taking (specifically statins and ACE inhibitors/angiotensin receptor blockers).

Standard publicly funded health benefits for seniors in Alberta provide them with premium‐free medication insurance coverage, but patients are required to pay a copayment of 30% of the list price of each medication, to a maximum of $25 CAD per prescription.^24^ The copayment elimination intervention was designed so that those randomized to this would have their copayments reduced to 0 for select high‐value CV preventive medications including the following: statins, beta blockers, ACE inhibitors, angiotensin receptor blockers, calcium channel blockers, diuretics, antiplatelet agents, anticoagulants, oral antidiabetes agents, insulin and smoking cessation aids. Other types of medications were not covered by this programme, neither were diabetes self‐monitoring supplies and other medical devices.

Participants who were randomized to the control arm of the trial received neither MOXIE nor copayment elimination. They continued to receive standard care through their regular health‐care providers and retained their usual copayment for medications.

### 2.2 Conceptual framework and study design

Two conceptual frameworks influenced the design of this study. First, the health belief model summarizes how and why people make health behaviour choices,^25^ and includes the constructs of perceived: susceptibility, severity, benefit and barriers—many of which can be targeted by SMES. Second, the framework proposed by Campbell et al^26^ postulates how financial barriers may affect clinical outcomes. These frameworks were used to inform the development of interview guides and preliminary coding templates. We used a qualitative descriptive study design to achieve the objectives of our process evaluation.^27^ Ethical approval was received from the University of Calgary Conjoint Health Research Ethics Board (REB13‐1241).

### 2.3 Sampling

Our target population for this qualitative process evaluation were trial participants (patients) and pharmacists who were involved in the recruitment process for the ACCESS study. We used separate purposive sampling strategies for patients and pharmacists. For each group, we defined several important characteristics that we wished to have represented in our final sample and contacted potential participants by phone. For patients, we anticipated needing to interview 30 individuals, though we planned to continue sampling until thematic saturation was achieved. Given that our objective was to receive feedback on the study interventions, we only sampled from those who received at least one intervention (i.e. not controls). We recognized that a variety of patient characteristics might affect their experience and perspectives on the interventions. The sampling considerations for participation in interviews included the following:

- Gender (men/women): gender differences have been noted in self‐management behaviour and perspectives.^28^
- Income ($≤30 000 vs $30‐50 000): those with lower incomes may stand to receive greater value from copayment elimination.
- Those who had perceived financial barriers on intake questionnaire: as above.
- Those who were not on indicated medications (i.e. ACE inhibitors/ARBs or statins) during enrolment: as those not on these medications were thought to be able to provide an important perspective on how the intervention impacted their medication use.
- Those who indicated less‐than‐perfect medication adherence during enrolment: as above.
- Type of intervention received (MOXIE, copayment elimination, or both): in order to ensure that we had perspectives of each intervention adequately represented.

For focus groups, we used a similar, but more limited, set of sampling criteria, including the following: gender, income, type of intervention received and method of recruitment (word of mouth/referral vs seeing recruitment material in a public space).

For pharmacists, we anticipated the need to interview 20 individuals and we used the following sampling strata:

- Urban/rural location: Pharmacists in rural locations often have more involvement in patient care than in busy urban pharmacies.
- Chain/independent pharmacy: as above.
- Gender (men/women): gender‐based differences in pharmacy practice have been noted.^29^, ^30^
- Those who had more significant involvement in recruiting for the study (i.e. recruiting 3 or more participants into the study) and those with less substantial involvement: those with more involvement were felt to be likely to provide more rich data, while those less involved could provide rationale for why they had not been more involved.

### 2.4 Data collection

Individual interviews were chosen as the principal means of data collection, as we were asking patients about how their personal situations changed as a result of the intervention. Patients were eligible to participate in an interview 6 months post‐enrolment. Given that our participants lived all over the province of Alberta, in‐person interviews were deemed to not be feasible. Therefore, we used semi‐structured telephone interviews^31^ to collect data from patients. Individual interviews were approximately 30‐60 minutes in duration. Interviewers were aware of some of the participants' responses from their baseline questionnaire (ie whether they were taking indicated medications), which helped tailor the questions that would be asked of participants in the interview.

Interviews were supplemented with 2 in‐person focus groups, held 1.5 years post‐ACCESS trial launch, which were conducted at the University of Calgary and lasted 2 hours. These focus groups were primarily focused on patients' recruitment experiences, and using this forum helped provide a richer discussion and to inform a relaunch of trial recruitment activities.

Additionally, we conducted telephone interviews with pharmacists, which ranged from 20 to 50 minutes in duration. The domains included in the interview and focus group guides for patients were (see Appendices A and B) as follows:

- Barriers to adherence/self‐management, and changes to these.
- General feelings towards the specific intervention they received (copayment elimination, self‐management education, or both).
- Whether the intervention had addressed financial and/or knowledge issues.
- Which aspects of their intervention had been helpful, and which had not.
- Why the intervention was successful at changing behaviour or not.
- What could be changed in the intervention to make it more helpful.

Pharmacists who helped recruit for ACCESS^22^ were also interviewed. Topics included in the interview guide for pharmacists were (see Appendix C) as follows:

- Familiarity with the ACCESS trial.
- Experiences with patients receiving the copayment elimination intervention.
- Experiences with patients receiving the MOXIE intervention, including the embedded facilitated relay intervention.

Patient and pharmacist interviews were conducted individually by two female research assistants who were trained in qualitative interviewing and had no prior relationships with participants. Interview guides were piloted with other members of the research team to ensure the questions flowed and the interviewers were comfortable with the sequence of questions. Focus groups were conducted by DJTC (male, Co‐Principal Investigator) and TSS (female, Research Coordinator) with other research staff present to help take notes. Participants were informed of the purpose of the interviews and focus groups. Notes were taken during all interviews and focus groups, and the proceedings were digitally recorded and transcribed verbatim by a professional transcriptionist.

### 2.5 Data analysis

Interview transcripts were imported into NVivo 11 software (QSR) to help organize the qualitative data. Analysis was undertaken by two independent reviewers (research assistant and TSS). Thematic analysis techniques were used to code the transcripts.^32^ We started with a preliminary coding template based upon the interview guides. Codes were added by each individual reviewer in an inductive fashion. The coders met to resolve discrepancies, involving a third reviewer when necessary (DJTC). The team met to amalgamate similar, granular codes into broader theme groups.

## 3 RESULTS

We conducted individual interviews with 39 patients (Table 1); 38 patients declined to participate in an interview. More of those interviewed were men with lower incomes and native English speakers (Table 1). In addition, we hosted 2 focus groups (n = 8 and n = 6); these patients were generally older and had lower incomes (Table 1). We approached 27 pharmacists for interviews, and 20 agreed to participate. As expected, the pharmacists had a relatively even distribution across the sampling criteria (Table 2). There were, however, slightly more men than women, and more pharmacists from rural settings than from urban centres (Table 2).

**Table 1:** Characteristics of patient participants

	Interviews (n = 39)	Focus group 1 (n = 8)	Focus group 2 (n = 6)
Intervention			
Copayment elimination	19	8	0
Self‐management education	24	0	6
Gender			
Woman	15	4	4
Man	24	4	2
Age			
65‐70	12	2	2
71‐75	12	2	3
>75	15	4	1
Income			
$0‐29 999	24	5	4
$30‐50 000	15	3	2
Marital status			
Married/common law	20	3	2
Single/other	19	5	4
Highest level of education			
Post‐secondary diploma or higher	14	3	4
Less than post‐secondary diploma	25	5	2
Number of people living in household			
1	12	5	2
≥2	27	3	4
Country of birth			
Canada	26	4	5
Other	13	4	1
Native language			
English	33	6	5
Other	6	2	1
Health literacy^a^			
Adequate	26	7	5
Inadequate	13	1	1

^a^ Using validated single‐item screening tool.^24^

**Table 2:** Characteristics of pharmacists (n = 20)

Characteristic	n
Recruiter significance^a^ Major: 11 Minor: 9	
Location^b^	Urban: 8
	Rural: 12
Type of pharmacy	Chain: 10
	Independent: 10
Gender	Female: 9
	Male: 11
Additional prescribing authority^c^ (unknown for one pharmacist)	Yes: 10
	No: 9

^a^ Major defined as recruiting 3 or more participants into the study.

^b^ Urban: Calgary, Edmonton, Red Deer, Lethbridge or Medicine Hat.

^c^ With special training and licensure, some pharmacists are eligible to prescribe medications.^23^

The three main topical areas covered by the interviews/focus groups were as follows: (1) feedback on the copayment elimination intervention, (2) feedback on the SMES intervention, and (3) feedback on the facilitated relay intervention embedded within the SMES. Further supporting quotes for each area and subarea are provided in Table 3. Identifiers are provided for each quote, which link to the participant tables (Appendices D and E).

**Table 3:** Patient and pharmacist feedback

Area	Quote
Copayment elimination	
Quality of life benefits	'*It is helpful, I notice a difference in what I pay for my bundle of medications that I get every three months… at least it gives me a little emotional boost' [Pt34]*. *'It really helps, I’m getting that little bit extra there for my other medications from your study… that makes managing everything easier' [Pt34]*. *'It just makes it that I don't feel like I’m penny‐pinching all the time' [Pt19]*. *'I don't have to worry about it. You, I just only have to worry about it, because there have been times that I’ve worried' [Pt8]*. *'It means I can buy a few more groceries, because I’m a very low‐income senior' [Pt29]*.
Direct health‐related benefits	*'If I hadn't [enrolled], I don't know what we would have done. Probably would have just stopped taking [the medications]. Some months I did have to take them every second or third day' [Pt38]*. *'[medication taking] has improved because I’m not paying for the cholesterol or the blood pressure medication, because what happens is that they all come due at the same time. I have COPD, so [that medication is] not covered obviously, so I’m paying that then I’d be paying for all the other stuff at the same time' [Pt14]*. '*If you guys pay a bit for my some of my medications then it gives me leeway. Because it gives me more leeway, I can also get my strips even though I have to pay fully for them, right?' [Pt1]* *'I mean [the amlodipine], that's the next thing I would have had to drop because you know…or the Lipitor but I mean I can do that now, with you guys paying for that I have an interest in taking it' [Pt1]*.
MOXIE feedback	
Knowledge	'*I like the tips on exercise or diet. They have links to different recipes…you know, the hints and tips and then there was the one that [highlighted] seniors places throughout Alberta. Different things and informative things like that' [Pt14]*.
Reminders	*'Like MOXIE will say, did you take the medication at the same time every day? That makes me think, oh yeah, it's time to take it now. Believe it or not a lot of [the improved adherence] is because of MOXIE' [Pt29]*. *'Somebody nudging you on the shoulder, "Hey, get with it!"' [FG1]* *'I think I’m paying more attention to it. I got pretty lax about because I was feeling pretty good and I wasn't staying on track with things. I am working to stay on track. I feel better and I feel like I’m in control of what I’m doing' [Pt8]*.
Pedometers	'*I’d put it back on my belt, but it just wouldn't stay on. So, when I mailed the last thing in, I had got the notice from you about how I could get a new Pedo‐thing' [Pt5]*.
Blister packing	'*They were like more comfortable in taking them [in vials], but I gave them the option' [Rx16]*.
Facilitated relay feedback	
Lack of provider interest	*'I just didn't think that it would add anything to our relationship or him looking after me… same with the druggist, he don't care' [FG1]*. *'I gave the one to the doctor, but he said, no that's alright, you keep it' [FG2]*. *'I don't even know because I just gave him the envelope and didn't really get a response from them' [Pt31]*.
Causing alarm	*'Yeah I figured if a person maybe wasn't up on their health or understood everything and they got that letter, it could panic some people' [FG1]*.
Inappropriate advice	*'In that particular individual it had been tried because there was [an] issue and that's the reason it was not in there, so we had not gone ahead with the recommendation' [Rx4]*.
Helpful reminders	*'Sometimes you know everybody can miss anything, so it's good to have another, you know, set of eyes looking at the stuff and making recommendation. It's totally good. It's good for the patient' [Rx6]*.

### 3.1 Area 1: copayment elimination feedback

Participants who received the copayment elimination intervention were overwhelmingly positive about the intervention. There were two main themes of positive feedback regarding this intervention: benefits to quality of life and direct health‐related benefits. Many participants described that the elimination of copayments benefited their quality of life, as it alleviated the need for budgeting closely: '*I have some extra to spend and it makes it easier to not have to budget quite as closely I did prior to that' [Pt16]*.

In addition to this benefit, several participants voiced that the elimination of copayments improved their health directly by alleviating pre‐existing financial barriers, allowing them to take their medications as indicated. One individual stated: '*Now I can go and get [my medications] when I need them, and I don't have to worry that I don't have the money. Because [before] I had to wait until I got my cheque' [Pt29]*. Pharmacists mentioned similar positive experiences with their patients.

I was in the process of formalizing deprescribing because he already stopped his statin medication. … but when he got approved [for the ACCESS trial], he was very happy. I talked to him, I explained to him that maybe it’s important to be on his preventative medications again and he accepted it happily, so we reinitiated his preventative medication. [Rx2]

The only negative feedback received about the copayment elimination centred on sentiments that the intervention did not cover enough medications or health supplies. Participants explicitly described that they wished the formulary would cover all medications (not just the high‐value preventive medications) and their diabetes testing supplies. However, even though these were not covered, some participants voiced that because some medications were free of charge, their adherence to non‐covered medications also improved and the cost savings enabled them to purchase health supplies and eat a healthier diet: '*Between the insulin and heart meds and all the rest of it, it really added up, and the food of course. If you are eating fruit and veggies, that's expensive at times too… But now it's just awesome. Being on this program has really helped. It means I can put more money on the fruit and veggies' [Pt31]*.

Pharmacists also noted patients were pleased with the intervention: '*We're waiving $32‐34 every time he is filling his prescription. That makes him happier as he's saving money towards his other needs. He's more compliant. He's not like really struggling with his medications or budget' [Rx3]*. Furthermore, some felt their ability to counsel patients was aided by copayment elimination '*because [patients were] not focused on the finances and therefore, [they] could concentrate on the medications' [Rx4]*.

### 3.2 Area 2: self‐management support programme (MOXIE) feedback

The MOXIE programme was received positively by most participants who received this intervention. The themes relating to the benefits of MOXIE include the following: (a) enhanced knowledge; (b) helpful reminders; and (c) improved confidence in self‐management ability.

One participant commented on how MOXIE provided information they were not aware of: '*I’ve picked up a few things from your email that are quite helpful. It is informative. I wasn't aware of all the things that are available for seniors' [Pt16]*.

In addition to education, MOXIE provided tips for remembering to take medications and some participants voiced that these tips, along with emails and reminders, helped them: '*I am having trouble remembering to take my medication. Something that I read in one of the MOXIE’s postcards, I ended up making notes, "remember your medications" and sticking them around the kitchen and everywhere. That really has helped' [Pt8]*.

The result of the improved knowledge and reminders was enhanced confidence and attitude regarding their chronic condition: '*I’ve got more confidence because somebody is writing to me… telling me what is good for me. That makes a little bit of difference to what attitude how one looks at things. You know we don't have to rely purely on the doctor. We can tell the doctor a little bit, which is good' [Pt15]*.

Despite the predominantly positive feedback, there was some negative feedback regarding MOXIE. The most frequent complaint revolved around the low quality of the pedometers provided. Additionally, participants mentioned that the education intervention was too medication‐focused, repetitive and/or not tailored enough. One participant stated: *'I don't feel that it's done me any good. The Moxie thing just keeps pushing statins and I know I cannot take them'* *[Pt5]*. Another participant commented: *'One of the things in the questionnaires that I answered, is that I am not very mobile. Many of the questions were centered on making sure you got your exercise. So, nobody has thought of people that are somewhat immobile' [Pt6]*.

As part of the intervention, participants were encouraged to speak to their pharmacists about medication reviews and blister packing. When asked how MOXIE may have helped them take medications, one participant stated: '*I guess how we take it [now] is this bubble pack… it's very helpful' [Pt25]*. Despite this explicit instruction from the MOXIE intervention, when we asked pharmacists, most had no recollection of any patients starting these discussions as a result of MOXIE. One pharmacist explained they may not have associated these conversations with MOXIE: '*We may have had a couple because out of the blue we had a couple start some blister packs that we probably didn't realize might have needed them' [Rx6]*.

### 3.3 Area 3: facilitated relay intervention feedback

Those randomized to MOXIE also received tailored letters, instructing them to take the letters to their family physician and pharmacist. The objective was to stimulate a discussion between participants and their health‐care providers about CV preventive medications they should ideally be taking. This portion of the intervention received mixed reviews. Some participants showed these letters to their health‐care providers (n = 15/24), while others did not (n = 9/24). In our focus group, only 1 of the 6 patients brought the letter to both their pharmacist and family physician. Of the 20 pharmacists interviewed, only 6 of them recalled receiving or reading this letter.

One participant commented on why they did not bring these letters to the health‐care providers: '*I just thought, these people are busy enough. I had discussions with them already. I would have felt embarrassed, I think, to take them in. I would have felt sort of foolish' [Pt13]*.

One patient provided a suggestion that might encourage patients to take the letters to their providers: '*Maybe if you had it explained in there like you explained it to us now, saying that you expected us to sort of go and give them extra knowledge, like you know just help them out in a way too. That might help' [FG1]*.

Of the 15 people who brought the letters to their physicians or pharmacists, eight said it did not prompt a medication discussion*: 'I just give him the letter and he just looked at it and put it in the file I guess' [Pt12]*. For some participants, the letters did lead to a discussion about their medications; however, most stated that it did not result in any changes: '*he looked at them and he said – no you are ok, you're ok, we will just leave these alone' [Pt4]*. One pharmacist mentioned that he had read the letter but decided against making the suggested medication changes. A few patients stated that the letters prompted a discussion, which resulted in starting a statin: '*I had to tell him, I think we should do it. So, then he gave me a smaller dose' [Pt15]*. Similarly, a pharmacist claimed that the letter pointed out a gap in patient care: '*I thought it was a good thing because there will always be someone you notice or maybe you don't that maybe they are not on all of the medications indicated for their condition. So, one of our guys wasn't on a statin and no one really knew why. You kind of assume that it's intentional, but it wasn't' [Rx11]*.

## 4 DISCUSSION

This qualitative process evaluation of the ACCESS trial implementation explored patient and pharmacist perspectives on the study interventions. The interventions (copayment elimination and/or a tailored SMES programme) were received positively and seemed to address the underlying barriers to chronic disease managment and CV risk reduction (financial difficulties and lack of education). Participants receiving the copayment elimination believed they derived both quality of life benefits (peace of mind and decreased worry) and health‐related benefits (improved medication, dietary and self‐monitoring adherence). Recipients of the self‐management education felt it provided them with trusted information and helpful reminders and improved confidence in their ability to reduce their CV risk. The facilitated relay intervention was less readily adopted as participants expressed hesitancy to take the letters to their providers. It is possible that the low level of health literacy in this sample (~50% of interviewees reported inadequate health literacy; Table 1) contributed to lower uptake of facilitated relay, as patients may have been hesitant to start a discussion for which they felt ill‐equipped. Some of those who did as the letters instructed had positive experiences, but the majority felt their providers were dismissive of the letters. Pharmacists’ responses largely mirrored those of participants—that both interventions were helpful to their patients, but they were much less aware of the educational intervention than we anticipated.

Reflexivity is an important element of qualitative research.^33^ We realized early on that participants’ candour in interviews may be limited if they perceived that this feedback was being collected by the study team as they may have felt that negative feedback could jeopardize their on‐going receipt of the trial intervention. To mitigate this view, we had an external team conduct the interviews who had no relationships with participants. However, the fact remains that there is a significant power differential between interviewers, as emissaries from the University, and study participants who were low‐income seniors with chronic health conditions—this may well have contributed to the predominantly positive feedback received. Throughout this process, the investigators of the randomized trial were able to reflect upon the feedback we were receiving. In response to this feedback, after approximately 1000 participants had been enrolled into the trial, the ACCESS trial interventions were significantly modified to address the concerns raised (Appendix F).

Our qualitative study found that the copayment elimination had the potential to confer benefits that were broader than those simply related to coverage of medications included on the study formulary. Reduced medication costs allowed participants to afford other health‐promoting goods and enhanced communication between patients and pharmacists, since patients were no longer as focused on money. This would suggest that potential differences in outcomes from this intervention may be related to these ‘side benefits’ in addition to the direct increased adherence to covered medications.

While important, it is clear that financial barriers are not the only reason that patients may be non‐adherent to medical therapies, given that adherence remains suboptimal, even in countries with full public pharmaceutical coverage.^34^, ^35^ Therefore, other interventions are also likely needed to enhance adherence. Self‐management education programmes can contribute to individuals’ health behaviours, including medication taking, in a variety of ways. Even though our intervention tried to provide tailored messaging, our results suggest that it would be strengthened by further and more detailed tailoring to patients’ individual needs and circumstances and avoid repetition. Clearly, participants felt that the messaging provided to them was not always tailored enough to be optimally useful. Changes to the messaging from MOXIE were made to improve acceptability (Appendix F). Furthermore, the unique strategies deployed in the MOXIE intervention were designed to provide participants with a sense of a relationship with the information source, which likely helped them feel more positive and engage with the material.

Previous studies, including high‐quality randomized trials, have demonstrated improved medication adherence^36^, ^37^ and clinical outcomes^8^, ^38^ for patients with CVD who receive interventions that reduce financial barriers, such as copayment elimination. However, none of these studies have utilized a qualitative implementation evaluation design to explore patients’ reception of the intervention, which might explain how and why it may have provided clinical benefits. Similarly, much research has demonstrated clinical benefits related to self‐management education and/or facilitated relay,^14^, ^39^, ^40^, ^41^ yet exploratory qualitative research into the reception of these techniques is largely lacking in published literature. This fits with a general call for qualitative methods to be used within interventional studies more frequently.^20^, ^42^

Our evaluation has limitations that merit discussion. First, the findings are subjective, and not all study participants have the same opinions of the interventions. However, given that we reached saturation in our sampling for both groups, we feel we have captured the breadth of perspectives on these interventions. Second, given that we intentionally chose to use a qualitative methodology, we cannot ascertain how prevalent positive or critical views of the interventions might be in the broader study sample; a quantitative study on this is currently underway. A strength of the study is that we explored perspectives of patients and pharmacists; however, we did not include physicians who may have received the facilitated relay letters, which would have added understanding of this perspective. Third, we largely explored the acceptability of the study interventions, which does not necessarily translate into altered patient behaviour (ie improved medication taking, diet and physical activity). These outcomes will be analysed in the on‐going clinical trial. Finally, this study was conducted after the intervention had been received, over a relatively short period of time. With this design, we cannot speak to the sustainability of the programme in supporting behaviour change in the longer term, which merits investigation.

## 5 CONCLUSIONS

The findings from this study provide insight into how interventions, like those being tested in the ACCESS trial, might work to help reduce patients’ CV risk. This work is important as there are numerous information technology firms working with health‐care organizations to provide digital platforms to patients to encourage healthy living. These qualitative findings will be bolstered by the findings of the trial, which will be able to answer whether adherence and clinical outcomes are improved as a result of these interventions. Future qualitative work is required to explore the experiences of people in clinical trials—particularly among individuals whose adherence/health behaviours are not improved through receipt of the study interventions.

## 6 Trial Registration Number

NCT02579655 (Clinicaltrials.gov)—initially registered October 19, 2015. URL: https://clinicaltrials.gov/ct2/show/NCT02579655?term=NCT02579655&draw=2&rank=1.

## CONFLICT OF INTEREST

Dr Tsuyuki has received investigator‐initiated grants from Merck Canada, AstraZeneca Canada, and Sanofi Canada. He has given paid presentations for Merck Canada and Sanofi Canada. These grants and presentations are unrelated to the current manuscript. Dr Pannu is the CEO and cofounder of EMERGENCE creative, who codesigned the MOXIE intervention. This manuscript has not been submitted for publication in any other journal.

## AUTHOR CONTRIBUTIONS

**DJTC** conceived of the study, acquired funding for the study, designed the study methodology, oversaw data collection and analysis, interpreted the results, wrote the first draft of the manuscript and edited the manuscript. **TSS** was involved in the conception of the study, assisted with data collection, performed qualitative analyses, and critically revised and edited the manuscript. **BJM, MT and BRH** obtained funding for the study, contributed to conceptualization of the study, assisted with interpretation of the results and critically revised and edited the manuscript. **NI, RT and RP** contributed to conceptualization of the study, assisted with interpretation of the results and critically revised and edited the manuscript. **KKS** assisted with conception of the study, acquired funding for the study, oversaw study design, data collection and analysis, interpreted the results, and critically revised and edited the manuscript. All authors read and approved of the final manuscript.

## Data Availability

The data that support the findings of this study are available from the corresponding author upon reasonable request.

## APPENDIX A

### PARTICIPANT FOCUS GROUP GUIDE

MOXIE focus group:

- Overall how has your experience been with MOXIE so far?
- What do you find to be the most helpful things about MOXIE?
- What could be improved to make MOXIE more helpful in helping you become your better self?
- Do you remember getting letters in your starter kit from Moxie? Did you take them to your Doctor/Pharmacist?
- Why or why not?

Copayment elimination focus group

- You are all receiving free medications. Has this helped you in the management of your chronic condition? If so, in what ways?
- What has the savings meant to you?
- What have you been able to do with the money that you otherwise would not have had?
- Have you experienced any problems with your coverage since joining the study?

## APPENDIX C

### PHARMACIST INTERVIEW GUIDE

1. Study knowledge
  Can you tell me what you know about the ACCESS trial?
  - What do you think the study investigators trying to find out/what is the objective of the study?
  - What are the interventions being used in the ACCESS study? (do they know anything about MOXIE?)
  - How does a participant enrol in the study?
  - What does participation entail? (do people have to travel to Calgary? Study follow‐ups)
  - How is one assigned to which intervention they are going to receive?
  - What outcomes do you think the ACCESS study team is going to follow?
2. Recruitment
  How did you hear about the ACCESS Study and what your initial interactions were like?
  - How was the ACCESS study explained to you?
  - Did you speak directly with a member of the ACCESS team? (Phone? Email? In person? Mail?)
  - In your opinion how did the conversation go?
  - Do you remember what your thoughts were initially?
  - Was the study explained to you clearly?
  Please describe the methods you used for recruiting your patients into the ACCESS study?
  - Calling patients directly
  - Speaking about the study to patients
  - Posters in pharmacy
    ○ How often did you replace the posters
  - Brochures on display in pharmacy
  - Putting brochures in prescription bags
    ○ How did you decide which patients to target for brochures
  - Full team at pharmacy aware of study?
  - Do you think that there are any major barriers to recruitment?
  - Have any patients mentioned the study to you?
  In your opinion, how have patients responded to the methods of recruitment you have used in your pharmacy?
  - How many patients do you think you attempted to recruit for the ACCESS Study?
3. The Educational Intervention (MOXIE)
  What do you know about the educational intervention of the ACCESS Study?
  - Have any of your patients ever spoken to you about MOXIE – or the cards they receive in the mail?
  - How do patients feel about these? Have you heard any feedback about them?
  Did any of your patients ever show you a letter in a blue envelope that they were asked to bring to their pharmacist?
  - Did you read this letter? (With your patient? After they left?)
  - What were your thoughts on the letter?
  - How did you feel receiving a letter from your patients about their medications?
  - Did any conversations about statins/ACE‐ARBs start because of this letter?
  - Did you ever fax the recommendations to the patient's prescriber?
  - Did any patients get started on one of these medications because of this letter?
  Have any of your patients who are enrolled in the study spoken to you about blister packing/medication reviews since they joined?
  - Did they mention whether this was recommended to them within the educational materials they received?
  - Do you think this might be beneficial for their care? How?
  Have any of your patients who are enrolled in the study who are smokers spoken to you about receiving support to help them quit smoking?
4. Copayment Elimination Intervention
  Have any of your patients been randomized to receive free medication coverage? What are your thoughts on the impact this has had on the patients?
  Have any of your patients had any trouble getting their coverage changed?
  - Have any of the medications that should have been covered, not been covered?
  - Did patients bring in the list of medications to be covered to review with you?
  - Were any participants surprised/upset that not all their medications, or their testing supplies were not covered?

In your opinion, what are patients’ perceptions of receiving free medications?

Have any patients mentioned the impact that this has had for them?

Did you find this was a conversation starter with your patients about their medications?

How do patients respond when they were not randomized to receive free medication coverage?

## APPENDIX D

### PARTICIPANT/QUOTE IDENTIFIERS, PATIENTS

**Table:**

Participant number	Age category	Sex	MOXIE (Y/N)	BC (Y/N)	Time between randomization and interview/FG (months)
Pt1	65‐70	Male	Y	Y	9.8
Pt2	>75	Male	N	Y	8.6
Pt3	>75	Male	N	Y	7.5
Pt4	>75	Male	Y	N	9.1
Pt5	>75	Female	Y	N	7.3
Pt6	65‐70	Male	Y	N	8.7
Pt7	65‐70	Female	Y	Y	11.0
Pt8	71‐75	Female	Y	Y	10.2
Pt9	71‐75	Male	Y	N	8.5
Pt10	71‐75	Male	N	Y	9.2
Pt11	71‐75	Male	Y	N	8.6
Pt12	71‐75	Male	Y	Y	8.2
Pt13	71‐75	Female	Y	N	8.6
Pt14	65‐70	Female	Y	Y	8.1
Pt15	71‐75	Male	Y	Y	6.6
Pt16	71‐75	Male	Y	Y	8.0
Pt17	>75	Male	N	Y	8.2
Pt18	>75	Female	N	Y	8.3
Pt19	>75	Female	N	Y	8.6
Pt20	>75	Male	N	Y	8.6
Pt21	>75	Female	N	Y	8.0
Pt22	>75	Female	Y	N	8.7
Pt23	>75	Male	Y	N	7.9
Pt24	>75	Male	N	Y	8.1
Pt25	>75	Male	Y	N	8.0
Pt26	71‐75	Male	N	Y	8.3
Pt27	71‐75	Female	N	Y	8.2
Pt28	71‐75	Male	Y	N	8.4
Pt29	65‐70	Female	Y	Y	7.8
Pt30	>75	Male	Y	Y	8.2
Pt31	65‐70	Female	Y	Y	7.8
Pt32	65‐70	Male	N	Y	8.2
Pt33	>75	Female	Y	Y	8.7
Pt34	>75	Female	N	Y	8.1
Pt35	65‐70	Female	Y	Y	8.0
Pt36	65‐70	Male	N	Y	8.6
Pt37	65‐70	Male	Y	Y	6.3
Pt38	65‐70	Male	N	Y	7.7
Pt39	65‐70	Male	Y	Y	7.0
Pt40 (FG1)	>75	Male	N	Y	7.3
Pt41 (FG1)	65‐70	Female	N	Y	9.9
Pt42 (FG1)	65‐70	Female	N	Y	9.4
Pt43 (FG1)	>75	Male	N	Y	9.4
Pt44 (FG1)	>75	Female	N	Y	5.8
Pt45 (FG1)	71‐75	Male	N	Y	4.4
Pt46 (FG1)	>75	Female	N	Y	3.6
Pt47 (FG1)	71‐75	Male	N	Y	1.7
Pt48 (FG2)	71‐75	Female	Y	N	6.0
Pt49 (FG2)	71‐75	Female	Y	N	6.2
Pt50 (FG2)	>75	Female	Y	N	4.3
Pt51 (FG2)	65‐70	Female	Y	N	2.5
Pt52 (FG2)	65‐70	Male	Y	N	3.3
Pt53 (FG2)	71‐75	Male	Y	N	2.7

## APPENDIX E

### PARTICIPANT/QUOTE IDENTIFIERS, PHARMACISTS

**Table:**

Pharmacist ID	Sex	Pharmacy Type	Location	Recruitment Significance
RX1	F	Chain	Rural	Major
RX2	M	Chain	Urban	Major
RX3	M	Chain	Rural	Major
RX4	F	Independent	Urban	Major
RX5	F	Chain	Rural	Minor
RX6	M	Independent	Rural	Minor
RX7	F	Chain	Urban	Minor
RX8	F	Chain	Urban	Major
RX9	F	Chain	Rural	Major
RX10	M	Independent	Rural	Major
RX11	F	Chain	Urban	Minor
RX12	M	Chain	Rural	Minor
RX13	F	Independent	Rural	Major
RX14	M	Independent	Rural	Major
RX15	M	Independent	Urban	Major
RX16	M	Independent	Rural	Minor
RX17	M	Chain	Rural	Minor
RX18	M	Independent	Rural	Minor
RX19	F	Independent	Urban	Major
RX20	M	Independent	Urban	Minor

## APPENDIX F

### CHANGES MADE TO STUDY INTERVENTIONS BASED ON FEEDBACK RECEIVED

**Table:**

Intervention	Feedback	Response
MOXIE/Self‐Management Education	Daily email contact was excessive	Email frequency was changed to 2 times per week
	Daily website cards/email were often repetitive	Additional content was created
	Messaging was too focused on medications	New content focused largely on health behaviours and other aspects of self‐management
	Paper‐based mailers were appreciated more than electronic communication	Electronic‐arm participants began receiving weekly mailers, just like paper‐based participants
	Pedometer quality was poor	Added a higher quality pedometer that participants received at the 1‐year mark. This upgraded pedometer was sent to participants as a replacement if their first pedometer broke
	Activity‐based messaging was not relevant to those with physical limitations.	More inclusive/less judgemental language was used in messaging around physical activity
Facilitated Relay	Patients were unclear what they were to do with the letters and why bringing these letters to their providers was important	A note was added to the exterior of the facilitated relay envelope, which provided details on the importance of bringing these letters to physicians and pharmacists
	Patients felt that their providers were not interested in the messages contained in the letter	Changes were made to the letter to highlight the importance for providers
Copayment Elimination	Some relevant cardioprotective medications were not automatically covered (when new additions were made to the formulary)	Procedures were put in place to review the drug formulary and add relevant medications to the study plan as they came onto the formulary
	Patients wished to have self‐monitoring supplies and other medications covered	No changes were made to address this concern
	Some patients were not entirely sure which medications were and were not supposed to be covered	Comprehensive and up‐to‐date medication lists were then sent to all new participants randomized to this intervention
	A small number of patients had problems getting their coverage through their existing plan.	We informed participants that our study team was available to help sorting out this problem, when necessary.

## References

[1] Bodenheimer T , Lorig K , Holman H , Grumbach K . Patient self‐management of chronic disease in primary care. JAMA. 2002;288:2469‐2475. 12435261 10.1001/jama.288.19.2469

[2] Lorig KR , Sobel DS , Stewart AL , et al. Evidence suggesting that a chronic disease self‐management program can improve health status while reducing hospitalization: a randomized trial. Med Care. 1999;37:5‐14. 10413387 10.1097/00005650-199901000-00003

[3] Bayliss EA , Ellis JL , Steiner JF . Barriers to self‐management and quality‐of‐life outcomes in seniors with multimorbidities. Ann Fam Med. 2007;5:395‐402. 17893380 10.1370/afm.722 PMC2000313

[4] Weaver RG , Manns BJ , Tonelli M , et al. Access to primary care and other health care use among western Canadians with chronic conditions: a population‐based survey. CMAJ Open. 2014;2:E27‐E34. 10.9778/cmajo.20130045 PMC3985957 25077122

[5] Campbell DJ , King‐Shier K , Hemmelgarn BR , et al. Self‐reported financial barriers to care among patients with cardiovascular‐related chronic conditions. Health Rep. 2014;25:3‐12. 24850391

[6] Campbell DJT , Manns BJ , Weaver RG , Hemmelgarn BR , King‐Shier KM , Sanmartin C . Financial barriers and adverse clinical outcomes among patients with cardiovascular‐related chronic diseases: a cohort study. BMC Med. 2017;15:33. 28196524 10.1186/s12916-017-0788-6 PMC5309998

[7] Campbell DJT , Ronksley PE , Manns BJ , et al. The association of income with health behavior change and disease monitoring among patients with chronic disease. PLoS One. 2014;9:e94007. 24722618 10.1371/journal.pone.0094007 PMC3983092

[8] Choudhry NK , Avorn J , Glynn RJ , et al. Full coverage for preventive medications after myocardial infarction. N Engl J Med. 2011;365:2088‐2097. 22080794 10.1056/NEJMsa1107913

[9] Tang KL , Barnieh L , Mann B , et al. A systematic review of value‐based insurance design in chronic diseases. Am J Manag Care. 2014;20:e229‐e241. 25326932

[10] Mann BS , Barnieh L , Tang K , et al. Association between drug insurance cost sharing strategies and outcomes in patients with chronic diseases: a systematic review. PLoS One. 2014;9:e89168. 24667163 10.1371/journal.pone.0089168 PMC3965394

[11] Bolle S , van Weert JC , Daams JG , , Loos EF , de Haes HCJM , Smets EMA . Online health information tool effectiveness for older patients: a systematic review of the literature. J Health Commun. 2015;20:1067‐1083. 26165846 10.1080/10810730.2015.1018637

[12] Peterson JC , Link AR , Jobe JB , Winston GJ , Marina Klimasiewfski E , Allegrante JP . Developing self‐management education in coronary artery disease. Heart Lung. 2014;43:133‐139. 24373484 10.1016/j.hrtlng.2013.11.006 PMC3947696

[13] Tun KS . Self‐management education in patients with cardiovascular disease: a best practice implementation project. JBI Evid Synth. 2014;12:454‐466.

[14] Barnason S , White‐Williams C , Rossi LP , et al. Evidence for therapeutic patient education interventions to promote cardiovascular patient self‐management: a scientific statement for healthcare professionals from the American Heart Association. Circ Cardiovasc Qual Outcomes. 2017;10:e000025. 28630370 10.1161/HCQ.0000000000000025

[15] Diabetes Canada Clinical Practice Guidelines Expert C , Sherifali D , Berard LD , Gucciardi E , MacDonald B , MacNeill G . Self‐management education and support. Can J Diabetes. 2018;42(suppl 1):S36‐S41. 29650109 10.1016/j.jcjd.2017.10.006

[16] Piepoli MF , Hoes AW , Agewall S , et al. 2016 European guidelines on cardiovascular disease prevention in clinical practice: the Sixth Joint Task Force of the European Society of Cardiology and Other Societies on Cardiovascular Disease Prevention in Clinical Practice (constituted by representatives of 10 societies and by invited experts) Developed with the special contribution of the European Association for Cardiovascular Prevention & Rehabilitation (EACPR). Eur Heart J. 2016;37:2315‐2381. 27222591 10.1093/eurheartj/ehw106 PMC4986030

[17] Royal Australian College of General Practitioners . General practice management of type 2 diabetes: 2016‐2018. 2016.

[18] O'Cathain A , Thomas KJ , Drabble SJ , Rudolph A , Hewison J . What can qualitative research do for randomised controlled trials? A systematic mapping review. BMJ Open. 2013;3:e002889. 10.1136/bmjopen-2013-002889 PMC3669723 23794542

[19] O'Cathain A , Goode J , Drabble SJ , Thomas KJ , Rudolph A , Hewison J . Getting added value from using qualitative research with randomized controlled trials: a qualitative interview study. Trials. 2014;15:215. 24913438 10.1186/1745-6215-15-215 PMC4059032

[20] Curry LA , Nembhard IM , Bradley EH . Qualitative and mixed methods provide unique contributions to outcomes research. Circulation. 2009;119:1442‐1452. 19289649 10.1161/CIRCULATIONAHA.107.742775

[21] Campbell DJ , Tonelli M , Hemmelgarn B , et al. Assessing outcomes of enhanced chronic disease care through patient education and a value‐based formulary study (ACCESS)‐study protocol for a 2x2 factorial randomized trial. Implement Sci. 2016;11:131. 27671037 10.1186/s13012-016-0491-6 PMC5037634

[22] Kakumanu S , Manns BJ , Tran S , et al. Cost analysis and efficacy of recruitment strategies used in a large pragmatic community‐based clinical trial targeting low‐income seniors: a comparative descriptive analysis. Trials. 2019;20:577. 31590686 10.1186/s13063-019-3652-5 PMC6781395

[23] Curran GM , Bauer M , Mittman B , Pyne JM , Stetler C . Effectiveness‐implementation hybrid designs: combining elements of clinical effectiveness and implementation research to enhance public health impact. Med Care. 2012;50:217‐226. 22310560 10.1097/MLR.0b013e3182408812 PMC3731143

[24] Campbell DJT , Manns BJ , Soril LJJ , Clement F . Comparison of Canadian public medication insurance plans and the impact on out‐of‐pocket costs. CMAJ Open. 2017;5:E808‐E813. 10.9778/cmajo.20170065 PMC5741433 29180377

[25] Rosenstock IM . The health belief model and preventive health behavior. Health Educ Behav. 1974;2:354‐386.

[26] Campbell DJT , Manns BJ , Leblanc P , Hemmelgarn BR , Sanmartin C , King‐Shier K . Finding resiliency in the face of financial barriers: development of a conceptual framework for people with cardiovascular‐related chronic disease. Medicine. 2016;95:e5561. 27930562 10.1097/MD.0000000000005561 PMC5266034

[27] Sandelowski M . Whatever happened to qualitative description? Res Nurs Health. 2000;23:334‐340. 10940958 10.1002/1098-240x(200008)23:4<334::aid-nur9>3.0.co;2-g

[28] Burner E , Menchine M , Taylor E , , Arora S . Gender differences in diabetes self‐management: a mixed‐methods analysis of a mobile health intervention for inner‐city Latino patients. J Diabetes Sci Technol. 2013;7:111‐118. 23439166 10.1177/193229681300700113 PMC3692222

[29] Carvajal M , Hardigan P . Pharmacists’ sources of job satisfaction: inter‐gender differences in response. Am J Pharm Educ. 2000;64:420‐425.

[30] Ip EJ , Lindfelt TA , Tran AL , Do AP , Barnett MJ . Differences in career satisfaction, work‐life balance, and stress by gender in a national survey of pharmacy faculty. J Pharm Pract. 2020;33:415‐419. 30518289 10.1177/0897190018815042

[31] Musselwhite K , Cuff L , McGregor L , King KM . The telephone interview is an effective method of data collection in clinical nursing research: a discussion paper. Int J Nurs Stud. 2007;44:1064‐1070. 16844128 10.1016/j.ijnurstu.2006.05.014

[32] Braun V , Clarke V . Thematic analysis In: Cooper H, ed. APA Handbook of Research Methods in Psychology. Washington, DC: American Psychological Association; 2012:57‐71. 701 pp.

[33] Dodgson JE . Reflexivity in qualitative research. J Hum Lact. 2019;35:220‐222. 30849272 10.1177/0890334419830990

[34] Grey C , Jackson R , Wells S , et al. Maintenance of statin use over 3 years following acute coronary syndromes: a national data linkage study (ANZACS‐QI‐2). Heart. 2014;100:770‐774. 24436219 10.1136/heartjnl-2013-304960

[35] Chen ST , Huang ST , Shau WY , et al. Long‐term statin adherence in patients after hospital discharge for new onset of atherosclerotic cardiovascular disease: a population‐based study of real world prescriptions in Taiwan. BMC Cardiovasc Disord. 2019;19:62. 30876393 10.1186/s12872-019-1032-4 PMC6420763

[36] Maciejewski ML , Farley JF , Parker J , Wansink D . Copayment reductions generate greater medication adherence in targeted patients. Health Aff (Millwood). 2010;29:2002‐2008. 21041739 10.1377/hlthaff.2010.0571

[37] Lewey J , Shrank WH , Avorn J , Liu J , Choudhry NK . Medication adherence and healthcare disparities: impact of statin co‐payment reduction. Am J Manag Care. 2015;21:696‐704. 26633094

[38] Kulik A , Desai NR , Shrank WH , et al. Full prescription coverage versus usual prescription coverage after coronary artery bypass graft surgery: analysis from the post‐myocardial infarction free Rx event and economic evaluation (FREEE) randomized trial. Circulation. 2013;128:S219‐S225. 24030410 10.1161/CIRCULATIONAHA.112.000337

[39] Shojania KG , Ranji SR , Shaw LK , et al. Closing the Quality Gap: A Critical Analysis of Quality Improvement Strategies (Vol 2: Diabetes Care). Rockville, MD: Agency for Healthcare Research and Quality; 2004. 20734526

[40] Seligman HK , Wallace AS , DeWalt DA , et al. Facilitating behavior change with low‐literacy patient education materials. Am J Health Behav. 2007;31(suppl 1):S69‐S78. 17931139 10.5555/ajhb.2007.31.supp.S69 PMC4286315

[41] Tricco AC , Ivers NM , Grimshaw JM , et al. Effectiveness of quality improvement strategies on the management of diabetes: a systematic review and meta‐analysis. Lancet. 2012;379:2252‐2261. 22683130 10.1016/S0140-6736(12)60480-2

[42] Nastasi BK , Schensul SL . Contributions of qualitative research to the validity of intervention research. J Sch Psychol. 2005;43:177‐195.

